# Russia’s Approach to Connectivity in Asia: From Cooperation to Coercion

**DOI:** 10.1007/s12140-023-09404-w

**Published:** 2023-05-01

**Authors:** Kristiina Silvan, Marcin Kaczmarski

**Affiliations:** 1 Finnish Institute of International Affairs (Russia and the EU’s Eastern Neighbourhood Research Programme), Helsinki, Finland; 2 School of Social and Political Science, University of Glasgow, Glasgow, Scotland, UK

**Keywords:** Connectivity, Russia, Eurasia, Foreign policy, Integration

## Abstract

Russia’s foreign policy concept, last updated in 2023, envisioned economic and political cooperation with countries of the Asia-Pacific as important for advancing Russia’s agenda as a global power and emphasised the need to improve connectivity across Eurasia. This article applies a novel theoretical framework for analysing Russia’s approach to connectivity in Asia since the collapse of the Soviet Union. Drawing from policy documents and secondary sources, the article identifies three different geographical spaces targeted by Russian connectivity policy: East of the Russian Federation, post-Soviet Central Asia, and Greater Eurasia. It is argued that the attempts to improve the cooperative connectivity of the Russian Far East have been half-hearted. In contrast, the attempt to retain and rebuild connectivity within the post-Soviet space has followed the logics of competition, containment, and coercion. Moreover, by promoting the Greater Eurasian Partnership, Russia has sought to keep status equality with China against the backdrop of the latter’s Belt and Road Initiative. The article maintains that Russia is a connectivity actor of its own right, even if there is a major gap between its connectivity strategy and its implementation. It further suggests that the war in Ukraine has accelerated the trend towards coercion and disconnectivity.

## Introduction: connectivity *po-russki*

Writing in September 2020, Andrey Kortunov, the Director General of the government-affiliated Russian International Affairs Council, lamented the fragmentation of Eurasia [24].^1^ In his manifesto for Eurasian integration, he argued that it would only be “natural” for the continent to “unite [...] in a single system, where different geographic components would organically complement each other” [24]. The emergence of such unity, he claimed, would be beneficial not only for those living in the Eurasian continent but for the rest of the world, too. Moreover, he argued that the construction of a unified space ought to be driven by economic means: both inter-regional trade and economic alliances and connecting integration projects. These pragmatic connections would serve as a basis of overall rapprochement, or — as he put it — “a lengthy historical project” also known as the Greater Eurasian Partnership (GEP) [24]. Contemporary Russia, targeted by unprecedented international sanctions for launching a war of aggression in Ukraine in February 2022 and declared a pariah state in the international community, seems to be lightyears away from living up to this vision.

In the twenty-first century world, connectivity has become the buzzword of policy parlance. There is a consensus that the term implies action that intentionally brings countries, people and societies closer together either by material or non-material means [22]. What is more, contemporary state and non-state actors are expected to be in principle interested in strengthening their connectivity with reliable partners, given that in a globalized world, being interconnected furthers development and modernization. China has been particularly active in framing its connectivity projects as a “win-win” for all those involved [8]. In practice, connectivity promotion has driven the emergence and evolution of various connectivity strategies. In the sphere of infrastructure, connectivity strategies currently in place range from China’s Belt and Road Initiative (BRI) to the US Blue Dot Network and the European Union’s Global Gateway. However, as Gaens, Sinkkonen and Vogt [18] point out in their theoretical contribution to this special issue, the concept is rarely defined with sufficient precision and risks becoming a mere empty signifier. They construct a broad analytical framework that identifies six logics of connectivity (cooperation, copying, cushioning, contestation, containment, and coercion), which play out differently within six different spheres (material infrastructure, economic, institutional, knowledge, people-to-people and security) [18]. The analysis of this article relates to five out of the six logics of connectivity: cooperation (i.e. the creation of inclusive mutually beneficial connective networks), copying (enhancing connectability by emulating others), contestation (gaining advantages over other actors through connectivity), containment (partial or complete exclusion of an actor through disconnecting or by building exclusive zones of connectivity) and coercion (forcing others to, connect a certain way or forgo certain connections altogether). The logic of cushioning (broadening the range of options through connectivity to mitigate risks) could be seen to play out in Russia’s attempt to diversify its energy exports away from the West, but given that Gaens, Sinkkonen and Vogt [18] see the logic mainly as a strategy of small or medium actors vis-à-vis great powers, its applicability in the Russian case is somewhat problematic.

In applying this framework to make sense of Russia’s approach to connectivity in Eurasia, the aim of this article is to contribute to the further development of the concept of connectivity and the theoretical framework constructed by Gaens, Sinkkonen and Vogt [18].^2^ Although Russia has not been the most vocal participant in the global connectivity debate, the article demonstrates that Moscow has been aware and concerned about the role of connectivity in the international system for decades. Given that the Russian economy depends on extracting and exporting natural resources like coal, gas, and oil to the global market, building and maintaining functional energy trade infrastructure has been in the Kremlin’s interest since at least the 1960s [35]. After the disintegration of the Soviet Union, Moscow was forced to readjust its connectivity strategy given the emergence of new state borders between the Russian Federation and the fourteen other post-Soviet republics. This article argues that for two decades, Russia pursued a two-track policy in its international connectivity related policy. It attempted to subordinate its post-Soviet neighbours as well as to retain and rebuild Soviet-era ties with a series of regional projects. Moscow also promoted the vision of “Greater Europe”, according to which western and eastern parts of Europe were supposed to function within a broader framework, with the European Union (EU) responsible for the former and Russia for the latter. Domestically, it sought to improve the connectivity of the eastern parts of the federation.

Russia’s quest for a great power status, the gradual worsening of its relations with the West, especially after the annexation of Crimea in 2014, as well as the rise of China and its promotion of the BRI have served as fertile ground for renewed connectivity considerations in the Russian Federation.^3^ The Eurasian Economic Union (EAEU), launched by Russia in January 2015, was expected to prevent further encroachment of the EU and China on the post-Soviet space. The GEP, officially announced in 2016, was to highlight Russia’s turn away from Europe towards Asia and to find a new role for Russia among mushrooming connectivity initiatives and regional projects in Asia.

This article applies the theoretical framework of Gaens, Sinkkonen and Vogt [18] on a diverse collection of policy documents and secondary sources to analyse the logics of Russia’s approach to connectivity, both domestically and internationally. It is situated in the debate discussing Russia’s declarative “pivot to Asia”, which underpins Moscow’s current Foreign Policy Concept, last updated in 2023 [14, 15]. In geographical terms, the paper focuses on Russia’s connectivity strategy in Central and Eastern Eurasia: in the Russian Far East (RFE), Central Asia, and within the so-called Greater Eurasia. It argues that in each of these three spaces, Russia’s attempts to foster connectivity follow a somewhat different reasoning. Improving the connectivity of the RFE is a means for regional development, while fostering connectivity in post-Soviet Central Asia with the help of institutions like the EAEU and the Collective Security Treaty Organization (CSTO) is an attempt to slow down the erosion of Soviet connectivity legacy. In turn, connectivity promoted in the framework of the GEP is meant to ensure symbolic status equality with China and counter connectivity frames promoted by the USA, such as the Indo-Pacific Region. What is highly distinctive about Russia’s approach to connectivity and can help to further develop this concept, in particular the role of agency, is the discrepancy between Russia’s ruling elite *thinking* about connectivity and activities undertaken by key Russian actors to *implement* connectivity ideas. As the case of China-Europe railway connections transiting the Russian territory demonstrates, the logic of containment which dominates the thinking about Europe stands in stark contrast to the logic of cooperation on the ground [Cf. 18].

By contrasting Russia’s different connectivity policies in Asia, the article develops three arguments. First, it suggests that although Moscow has no uniform strategy for connectivity, it is involved in connectivity initiatives at home and abroad. Second, due to Russia’s limited resources and apparent lack of political will, its connectivity practices reflect primarily political rather than economic reasoning, thus reflecting the political realities of contemporary Russia. This is why the development effect on the ground remains limited [31, 32]. Russia’s ongoing war in Ukraine, fully launched in February 2022, has only strengthened this trend and accelerated the trend towards *dis*connectivity. Third, as our contribution to the framework by Gaens, Sinkkonen and Vogt [18], we argue that the case of Russia demonstrates the co-existence of different logics of connectivity at play with regard to both different regions and vis-à-vis different actors. Whereas Russia’s connectivity projects towards its neighbourhood and the West are located towards the coercion end of the spectrum constructed by Gaens, Sinkkonen and Vogt [18], its approach towards China and Chinese projects represents the logic of cooperation. In addition, the logic of copying is present in a number of Russian connectivity initiatives across different spheres.

### The Russian Far East: Half-Hearted Connectivity for Development’s Sake

The administrative region called the Russian Far East (RFE) comprises 41% of Russia’s entire territory in the east. It borders with China and North Korea, and only a small strip of water separates the Sakhalin Island from Japan and Chukotka peninsula from the USA (Alaska). Due to its strategic location, it is important from the security perspective and has indeed been characterized as “highly militarized” [9: i]. The RFE is also significant due to its natural wealth, given that it is rich in gas, oil, minerals, diamonds, gold, tin, and other resources. However, the RFE is marginal in terms of its population: its six million inhabitants are poor and sparsely scattered around the region. What is more, the region is very distant and poorly connected to Moscow and Western parts of Russia. Following the collapse of the Soviet Union, the RFE has been suffering from a lack of systematic engagement. Despite the promising natural wealth, the connectivity infrastructure of the RFE is notoriously underdeveloped [13]. Rather than becoming a gateway to Asia, the region has turned into a “double periphery” — of Russia and of East Asia [26]. In a vicious circle, prospects for economic development continue to depend upon the improvement of the physical infrastructure: roads, rail lines, air routes, bridges, and pipelines. At present, the number of paved roads is still limited, there are just two rail lines, and very limited pipeline capacity [9: 59, 9].

Russian policymakers both in the Kremlin and in the region regularly voice concerns about the gap between potential and current level of connectivity. In 1987, Gorbachev announced a plan for the long-term development of the Soviet Far East to the year 2000 [26: 481]. During his electoral campaigns in the 1990s, President Boris Yeltsin launched a regional development programme aimed at “radically improving” the economic situation in the region. Yet the promised funding and investment never materialized [9: 62]. During Dmitrii Medvedev’s presidency (2008–2012), the RFE was envisioned as the gateway for Russia’s “pivot to the East” on the ground. One of the symbolic signs of the RFE’s elevated status and future development was the organisation of the Asia-Pacific Economic Cooperation (APEC) summit in Vladivostok in 2012. However, there were again no tangible and durable effects on the ground in the summit’s aftermath.

The discussion about the RFE’s modernization is also linked to debates about the relevance of Asia for Russia’s foreign policy. As Kuhrt [26: 473] points out, “Russian policy makers tend [… ] to link Asia-Pacific regional policy specifically to the development of the Russian Far East and vice versa”. Speaking in 2012, President Vladimir Putin voiced the hope that Russia could “catch some of China’s wind in the sails of our economy”, and thus accelerate mutual investment and technology transfer [Putin, quoted in 13: 5]. Following the gradual worsening of Russia’s relations with the West, particularly after the 2014 annexation of Crimea, Russia’s foreign policy has witnessed a gradual reorientation towards Asia. In contrast to Russia’s 2013 Foreign Policy Concept, which made only passing reference to collaboration with Asian partners, the 2016 edition underlines Moscow’s desire for closer economic and political collaboration with the Asia-Pacific region. Asia-Pacific’s “dynamic integration” is portrayed as an opportunity for Russia to implement its own domestic programs of socio-economic development in Siberia and the Far East [14: Point 78; see also 12]. The 2023 Foreign Policy Concept does not mention the Far East but speaks instead about “the strengthening of economic and transport connectivity in Eurasia” [15: Point 54].

The necessity to strengthen the connectivity between the Russian Far East and the Western parts of the Russian Federation is well pronounced, too. As a border region, the RFE has a pivotal role in the discourse on the sovereignty and territorial integrity of the Russian Federation [26]. In the 1990s, the RFE was led by secessionist governors threatening to break away from Moscow if the centre overlooked their interests [26: 477]. Writing in 2003, Davis [9: 1] suggested that due to their geographical location, “many in the RFE felt and feel that their future is tied to that of Asia and yet their present is tied to Moscow”. Indeed, Amirov and Mikheev [3: 62] argued in the early 2000s that the region had become economically highly dependent on Asia, which had alarmed the policymakers in Moscow. The Minister for Regional Development, Viktor Basargin [quoted in 26: 478], argued that the federal government ought to provide transport subsidies to businesses in the RFE to prevent the region’s economy from becoming “cut off” from the “European” part of Russia. Calls to strengthen the RFE’s connectivity with China — across various spheres ranging from people-to-people connections to trade — have been particularly securitized and politicised [26]. The assumption is that Chinese connectivity in the RFE could follow a logic of contestation over the central authorities in Moscow.

However, the RFE has not witnessed a considerable improvement of its connectivity with the Western parts of Russia. The lack of progress is particularly puzzling given that the development of the RFE has since 2013 been personally, systematically, and explicitly endorsed by President Putin and promoted by a separate specialized ministry, the Ministry of the Development of the Far East and the Arctic (abbreviated *Minvostokrazvitiya*) [42]. The Ministry has the function of coordinating the plethora of state and federal target programmes in the Arctic and the RFE [33], but as Savchenko [42] and Minakir [31] argue, the fundamental weakness of the organ has contributed to its inability to achieve its target growth rate or development quality in the region, despite its claims to the opposite. For now, the aims of the *National Programme of Socio-Economic Development of the Far East* [34] remain unfulfilled, too [31].

From the perspective of connectivity, improving the region’s road, rail and airport connectivity infrastructure would serve an important social function and bring the domestic population together. It is no coincidence that during his address at the 2021 Eastern Economic Forum, President Putin noted the necessity to expand the capacities of the Baikal-Amur Mainline and Trans-Siberian Railway [25]. Yet the policymakers have demonstrated that they prefer politically important projects over those of practical use: for example, funds from a bridge-building project across the River Lena in Yakutsk were redirected in 2014 to support the construction of the Crimea Bridge in Ukraine (on the profound symbolic and performative connotations of launching new transport corridors in the Russian context, see Pynnöniemi [38]). Meanwhile, the RFE has failed to develop into the planned trade and investment hub connecting Asia with Russia (and Europe). According to Ferris and Connolly [13], the region is stuck in a vicious circle: it needs (foreign) investment to improve its inadequate rail and sea transport infrastructure, but is unable to attract it due to its underdevelopment (caused by issues ranging from corruption to government hesitation, discussed above). As the final section of this article elaborates, the sanctions placed on Russia after the 2022 invasion in Ukraine will make foreigners even less likely to invest in Russia. Japan, South Korea, and Taiwan have joined the “collective” Western front of sanctions against Russia.

The sanctions are also further complicating domestic and international air travel. In the *Transport Strategy of the Russian Federation* [49], air *dis*connectivity of the RFE and the Arctic are elaborated in detail. The strategy points to the barriers that high fuel prices, reflected in the price of flight tickets, puts on citizens’ mobility, the existing programmes of subsidization notwithstanding [49: 42, 119]. The Strategy [49: 43] also points to the prevalence of foreign leased aircraft (catering 85% of civilian flights), which in the current context of Western sanctions has caused vivid debate on the plight of Russian aviation [2]. The former cost-benefit challenge of finding a balance between air travel subsidizing and the profitability of air carriers has been taken over by much fundamental problem of ensuring citizens’ with (safe) aviation services in any form, whether inside Russia or between Russia and the rest of the world [48]. Against this backdrop, the rise of cooperation with China is the only bright point. In June 2022, the first road bridge via the Amur River, between Blagoveshchensk and Heihe, was open [39]. The bridge was completed two years earlier, but the Covid-19 pandemic delayed the opening (on the importance of ceremonial openings in Russia, see Pynnöniemi [38]). The railway bridge, linking Nizhneleninskoye in the Jewish Autonomous Region and Tongjiang in Heilongjiang province, was completed in 2021 but its opening has yet to take place [19]. But even those achievements bleak given that it took more than 25 years to build the very first rail and road bridges between Russia and China. The bulk of evidence suggests it was Moscow that posed regular obstacles.

What is more, Russian policymakers have, in fact, obstructed the establishment of closer links with Asia and halted investment to the RFE. While Japan’s territorial grievances towards the Southern Kuril Islands (Northern Territories according to the Japanese nomenclature) remain the key obstacle to Russo–Japanese rapprochement, Tokyo attempted to find a formula allowing for closer cooperation, an ambition in the 2010s driven primarily by geopolitical concern of growing Sino-Russian ties. Abe Shinzo pursued a consistent policy of engaging Russia as of 2014. In 2016, he offered a set of initiatives directed towards the RFE and prepared on the basis of Putin’s annual addresses. This 8-point policy proposal included among other things healthcare, urban infrastructure, and agriculture [4]. However, the Russian side did not respond to those initiatives, having increased political-military pressure on Japan instead. This seems to indicate the predominant worldview of the Russian ruling elite when it comes to the RFE development — geopolitical priorities and concerns tend to take precedence over practical improvements in the region’s connectivity. The Japanese side must have taken Putin’s regular airing of concerns about socio-economic difficulties faced by the RFE seriously, hoping to offer targeted and local needs-oriented investment. From Moscow’s perspective, any meaningful rapprochement with Japan would have to lead to the weakening of Japanese–American ties.

In effect, the decade-long deprioritization of the RFE by the central government has fuelled a sense of *dis*connectivity from Western Russia. In 2020, resentment towards the Kremlin triggered week-long protests against the removal of Khabarovsk’s popular governor, Sergei Furgal. Moreover, the sense of disconnectivity seems to be somewhat mutual: although the Putin administration typically violently suppresses anti-regime protests at an early stage, the protests in Khabarovsk were allowed to go on for months. Makarychev [30] suggests that it was because the demonstrations in Khabarovsk were less dangerous for the Kremlin, given their physical distance from Moscow and the lack of explicit criticism of Putin’s foreign or domestic policies.^4^ This is not to say that the central authorities would be indifferent to events in the RFE — as this section highlights, the region’s development and connectivity to the Western parts of Russia is arguably seen to be of national importance. In the 2017 *Strategy for the Development of the Information Society* [47], for example, the further development of information and communications infrastructure is spelled out as a factor ensuring national security.

### Central Asia: Retaining and Rebuilding Soviet Era Connectivity

Until the establishment of the maritime routes between Europe and East Asia, Central Asia was located at a crossroads of major trade routes. The “silk roads” connecting Asia and Europe passed through the region, propelling development of Central Asia [16]. Centuries later, the territories of the five Central Asian states — Kazakhstan, Kyrgyzstan, Tajikistan, Turkmenistan, and Uzbekistan — were incorporated into the Russian Empire step by step. The northern grasslands of the region came under Tsarist rule already in the 1730s, while the annexation of settlements in the south occurred in the 1860–80s. The territories of Khiva and Bukhara were only incorporated into the Soviet Union in the 1920s. The imperial conquest was not motivated by a conscious desire to acquire territory and to assimilate its inhabitants, but rather by the ongoing competition with Great Britain and the ease of advancing into the region [1]. During the Soviet era, Central Asia was fully incorporated into a part of the Moscow-led empire. While Cold War scholarship viewed Central Asia as a colony of Soviet Russia par excellence, later work has sought to integrate colonialist explanations with frameworks of Soviet socialist modernity, modernization, and development [23].

The effect of Central Asia’s incorporation into the USSR on the ground was undisputed. In the twentieth century, the region became a part of the Soviet economy, planned as a single unit. Central Asia’s role in this economy was to supply raw materials — cotton, minerals and energy. Each republic specialized in producing a specific agricultural commodity. Like in other parts of the Soviet Union, agriculture was forcibly collectivized in the 1930s. The transport network, set up in the twentieth century, was designed to connect peripheral republics to Russia. Railway lines, roads, and oil and gas pipelines all connected Central Asia with Western parts of Russia. People-to-people connectivity was directed towards Moscow as well, with the dominance of the Russian language and, to an extent, the Russian culture, was established over time. Meanwhile, other directions of connectivity were restricted. After the Sino-Soviet split of 1960, the border with China was sealed and cross-border interaction was limited [37: 3–9].

Upon the break-up of the Soviet Union, Central Asian states were first and foremost linked with the Russian Federation in all six spheres of connectivity identified by Gaens, Sinkkonen and Vogt [18], namely infrastructure, economy, governance, knowledge exchange, culture, and security. What is more, Russia was initially eager to *disconnect* with the region that had been portrayed as a burden slowing down Russia’s development. For example, Yeltsin delivered a major shock to Central Asian economies by pushing Kazakhstan, Uzbekistan, and Turkmenistan out from the rouble zone in July 1993 [7].

The initial “post-imperial” withdrawal did not last for long, however. Russia took a more assertive stance towards states in its “near abroad” already in mid-1990s [40]. Clunan [5] argues that a consensus about the country’s role as a global great power and the rightful hegemon in the post-Soviet space emerged among the Russian elite as early as 1994. What is more, Yeltsin concluded early on that “ignoring” the post-Soviet region could have a negative impact on Russia’s own security [37]. As a result, Russian leadership adopted an approach whose aim was to preserve the existing level of connectivity in Central Asia. However, the policy was a rather passive one. The 1992 Tashkent Treaty of Collective Security laid the basis, first, for Russia’s security presence in the region, and, second, the emergence of the Collective Security Treaty Organization in 2002. In the sphere of economics, countries of the Commonwealth of Independent States (CIS) agreed on the establishment of the CIS Economic Union (1993), the CIS Free Trade Area (1994), the CIS Payment Union, and the first Customs Union (1995). In 2000, Belarus, Kazakhstan, Kyrgyzstan, Russia, and Uzbekistan established the Eurasian Economic Community (EurAsEC) [17]. Although anti-Russian sentiment and narratives about Russia’s colonialism were on the rise in Central Asia in the early 1990s, they were marginalized by the beginning of the 2000s [27].

After Putin’s ascension to presidency in 2000, Moscow has been increasingly eager to preserve the existing level of connectivity between Russia and the Central Asian states, and strengthening it when possible. The prevailing logic of Moscow’s connectivity strategy across all six spheres identified by Gaens, Sinkkonen and Vogt [18] seems to be either contestation or containment with coercive potential. Russia’s aim to act as a regional hegemon in Central Asia is by definition exclusionary vis-à-vis other actors seeking to increase their influence in the region. However, given Russia’s rapprochement with China, its connectivity strategy in spheres where China is also making headway leans more on contestation than containment.

Take, for example, the infrastructural sphere of connectivity [18]. The essence of Moscow’s policy from mid-1990s onwards has been to prevent the emergence of alternative infrastructural links, especially those that would bring natural resources from Central Asia to the European market. For years, Russia has opposed projects designed to reduce Europe’s dependency from Russian natural gas. The most famous of these, the Nabucco pipeline, failed in 2013 [44]. There were also attempts to increase Russia–Central Asia connectivity in the sphere. In 2007, Russia succeeded in convincing Kazakhstani and Turkmenistani leadership to construct a new trans-Caspian gas pipeline that would bring Turkmenistani gas to Russia. The agreement was never implemented, though.

Russia’s monopoly over Central Asian infrastructure was nonetheless gradually undermined, most consistently by China. Beijing began by opening a railway connection with Kazakhstan in the early 1990s. In the second half of the 2000s, China constructed an oil pipeline from Kazakhstan and a gas pipeline from Turkmenistan (via Uzbekistan and Kazakhstan). In the 2010s, China replaced Russia as the main export destination for Central Asian gas [36]. Yet especially Kazakhstan remains dependent on Russia for its energy exports, given that the Caspian Pipeline Consortium traverses through the Russian port of Novorossiysk on the Black Sea. Although the overall logic of connectivity is collaborative, Russia has exploited Kazakhstan’s (and Europe’s) dependency on the pipeline. After the beginning of Russia’s 2022 aggression in Ukraine, oil exports have been temporarily interrupted five times, triggering Astana to explore alternative trade routes to Europe [45].

In the sphere of economic and financial exchange, Russian connectivity strategy follows a similar logic that is contesting vis-à-vis China and containing vis-à-vis the West. The Russian leadership decided to pursue a more active response in the aftermath of the global economic crisis of 2008. Drawing on the experience of past failures in establishing closer economic cooperation within the post-Soviet area as a whole, Russia chose to pursue integration in the narrowest possible format, with Belarus and Kazakhstan as partners. In 2010, the new Customs Union was launched among the three and yielded way for a more ambitious integration project. When Vladimir Putin proposed the idea of the Eurasian Union in late 2011, Minsk and Astana were again selected as the key partners. The Eurasian Union was supposed to reinvigorate Soviet-era ties, consolidate Russia’s position in the post-Soviet space and prevent further loss of influence in the region to China and the West. From the outside, all the integration projects of the 2000s and 2010s appear to follow the “copying” logic of connectivity, identified by Gaens, Sinkkonen and Vogt [18], mimicking European institutions [17].

However, the idea of a Eurasian Union did not receive the support that Putin had envisioned. The 2013–2014 Revolution of Dignity in Ukraine, followed by the Russian annexation of Crimea and support to separatists in Donbas made Ukraine’s participation in any Russia-led connectivity projects politically impossible (until then, even when Ukraine formally endorsed participation in such initiatives in order to gain an upper hand in domestic power struggle, it was in practice slowing down their implementation). Moreover, Russia’s hostility towards Ukraine securitized and politicised Russian connectivity projects in other parts of the Former Soviet Union. As a result, both Belarus and Kazakhstan forced Moscow to limit the integration to the economic domain, which led to the establishment of the EAEU. In addition, the power of the EAEU’s attraction has turned out to be limited. Given its “hub and spoke” model and the asymmetry of the members’ markets, the EAEU has for now been considerably more useful economically to Russia. Ukraine’s engagement with Russia over the EAEU in 2013 also demonstrated that membership is mutually exclusive with that of the EU, pointing at Russia’s containing logic of connectivity. While Armenia and Kyrgyzstan joined the organisation relatively quickly, more powerful actors, such as Uzbekistan, have opted out of the organization, even if they would arguably gain some benefits from accession [20]. What is more, the EAEU is not the only element of Russia’s connectivity strategy in Central Asia. Russia remains the second biggest trade partner (after China) for all of the countries except Turkmenistan. Both informal and formal linkages within the business community are strong, also in strategic sectors. Especially the connectivity generated by the presence of Kyrgyz, Tajik and Uzbek labour migrants in Russia can be utilized by Moscow for coercive ends. In 2014, Russian authorities threatened to expel many of its Central Asian migrant workers if those states supported the UN resolution condemning the annexation of Crimea.

To conclude, Russia’s connectivity strategy in post-Soviet Central Asia is driven by hegemonic aspirations [40] that, in turn, leads to the dominance of contesting, containing and coercive logics. The primacy of (geo)political goals continues to limit Russia’s prospects of implementing its regional cooperation projects, although Moscow has been relatively successful in ensuring the survival of Central Asia’s authoritarian and kleptocratic elites [6]. All in all, Moscow seems to be interested in the instrumental use of the EAEU for its political purpose of elevating its great power status to the detriment of practical implementation. What is more, protectionist impulses and the lack of adequate financial resources dissuade Russia from a genuine pursuit of regional integration, which would incur serious costs next to potential benefits [17]. The most successful element appears to be the functioning labour market within the EAEU, which allows citizens of member states to work legally in Russia. In addition, in the context of the ongoing Russo-Ukrainian war, the connectivity in the spheres of passenger transport infrastructure and cross-border governance (i.e. visa-free movement) that primarily serves circular migration to Russia was utilized by hundreds of thousands of Russians fleeing the draft.^5^ While there is no reliable data on how many Russians are staying in Central Asia, the presence of these recently relocated migrants and companies is a factor that can strengthen the connectivity between Russia and Central Asia in some of the spheres identified by Gaens, Sinkkonen and Vogt [18], even if the general trend is towards *dis*connectivity [21].

### Greater Eurasia: Cooperation, Contestation and Containment

Two approaches have stood out in Moscow’s connectivity policies beyond the post-Soviet space: one that identified Russia as a link between Asia and Europe, and another that envisioned the creation of a distinct super-region of Eurasia. The former has been driven by practical and mercantile considerations; the latter by power-political ones. The practical and economically profitable reasons dictated the use of Russia’s vast territory as a land bridge between flourishing Asian economies and their counterparts in Europe. This logic also explains Russia’s push towards making the Northern Sea Route (NSR) navigable all year round, including the construction of necessary infrastructure and the promotion of the NSR as an alternative maritime connection between Asia and Europe. Power politics implied the use of connectivity as an instrument of great-power politics and status-seeking. Whereas the geopolitical type of thinking can be traced back to the ideas of “Greater Europe” of the early 1990s, the emphasis on Eurasia was ‘forced’ upon Moscow by growing activities in the connectivity realm of its peers, China in particular. In the case of China, Russia found it difficult to prevent the development of connections between China and Europe. Friendly ties with Beijing pushed Moscow towards acceptance of the Chinese version of the Eurasian transit bridge, which still allowed Russia to keep its central place. The lack of own manufacturing basis in turn meant that Russia was unable to fill in the connectivity potential with its own products.

As a consequence, beyond the post-Soviet space, Moscow is torn apart between a more cooperative logic of connectivity that dictates joining other players and participating in their efforts to develop new links throughout the region on the one hand and trying to “carve out” space for the Russian regional project which would at the very least limit the presence of other external players on the other and make Russia a major actor in this regard. The very shape of the Russian political-economic system generates a certain degree of inconsistency. Major players in Russia’s connectivity business, such as oil and gas producers, the Russian Railways (RZhD) or Rosatom, are either state-owned companies or private entities with close ties to the Kremlin. Their parochial commercial interests that they are able to lobby for, push Moscow towards a cooperative logic of connectivity. Geopolitical aspirations and the search for affirmation of its great-power status favour a competitive/coercive logic of connectivity.

### The Logic of Cooperation

The ideas of Russia playing a major role in linking Asian and European markets have been centred around the revival of the Trans-Siberian Railway (TSR) and the railway freight. This route was originally employed by Japan for shipping containers to Europe and the Middle East since the 1970s, but it lost its popularity towards the early 1990s. South Korean companies partially replaced their Japanese counterparts, but the so-called Siberian Land Bridge continued to lose to competitive pricing of maritime routes. The route experienced a partial revival in the 2000s, but this time it was China — and its north-eastern provinces — that dominated the transit with 70% of containers, followed by South Korea (20%) and Japan (almost 10%).

Russia’s situation and prospects changed in the early 2010s when China began opening railway connections with the European Union states. The majority of those connections operate on Russian territory and employ the western part of the Trans-Siberian Railway, with the entry point for transit on the Kazakhstan-Russian border. In the post-Soviet space, the route is operated by the joint Kazakhstani-Russian-Belarussian railway company, United Transport and Logistics Company (UTLC). In 2021, as many as 15,000 trains used the China-EU railway route carrying almost 1.5 million TEUs. As a result, the Chinese initiative has become a direct competitor for the Russian-led freight from Vladivostok. Additionally, the route from China to Kazakhstan generates competition for the Central Asian markets, which now can be accessed using both the Trans-Siberian railway and the route from China. The establishment of the regular railway connection between China and Europe was mostly driven by commercial interests of economic actors, including stakeholders from Russia (RZhD), with the governments playing a secondary role. This fact sheds light on the inconsistency of Russian policy in the sphere of connectivity.

The accelerating climate change has paved the way for another major connectivity project, the NSR. Moscow aims to provide necessary infrastructure — ports, icebreakers, piloting schemes — which would enable it to benefit from shortening the maritime route from Asia to Europe. So far, the NSR has been used only on a partial basis.

The NSR illustrates similar inconsistencies in Russia’s approach to connectivity as other initiatives. Russian elites seem to be divided between the proponents of maximising the use of the Arctic for economic reasons (for which the construction of infrastructure along the NSR is the main priority) and those perceiving the Arctic as the “ultimate frontier,” sovereignty over which should be guarded against everyone, including even friendly states such as China. It is commercially oriented actors such as state-owned Rosneft or the private company Novatek that follow the logic of cooperation, pushing for the extensive support for the NSR.

#### The Logic of Contestation and Containment

China’s Belt and Road Initiative (BRI), launched in 2013, has become an even bigger political challenge for Russia. The project was a gigantic leap in geopolitical and geoeconomic imagination, transcending traditional political boundaries. While not aiming explicitly to bypass Russia, the BRI put China in the position of an ‘organiser’ of the Eurasian space. Beijing’s project gained an almost global recognition and became the trademark of both a risen China and its leader, Xi Jinping.

The power-political dimension seems to have preoccupied the Russian elite much more than the search for practical solutions to growing competition from corridors established by China and from maritime transport. Moscow responded to the BRI by proposing the Greater Eurasian Partnership. In its grandiose scale, the Russian initiative envisions a network of connections between key Asian powers — Russia, China, India — and regional organisations, from the Shanghai Cooperation Organisation (SCO) and BRI to ASEAN. Moscow aimed to capitalise on already existing connections and make Russia a hub linking regional groupings from different corners of Eurasia. Whereas driven by power-political considerations and aimed at matching China’s initiative, the Greater Eurasia project espoused the logic of cooperation rather than competition. As the Russian idea has been directed towards great powers and small states alike, following a coercive pattern has not been a feasible option. The only actor effectively shut off from the project has been the USA. Greater Eurasia was supposed to serve as a testimony of Russia’s capacity to rival the USA and forge its own regional project.

Although Moscow portrays Greater Eurasia as a comprehensive connectivity project, it neither seems to be interested in its practical implementation, nor has financial and administrative capacity to pursue such an idea. Still, Beijing’s recognition of Greater Eurasia as a legitimate counterpart of the BRI meant that Moscow’s primary objective has been secured — the façade of equality vis-a-vis China.

#### Connectivity in the Greater Eurasia and Russia’s War in Ukraine

While formally open to European states, the concept of Greater Eurasia symbolised Moscow’s shift towards partners *beyond* Europe and constituted a response to the tensions generated by the annexation of Crimea. Conceptually, Russia chose to increase its connectivity with the non-Western world [28]. This shift has never been absolute, however, and the practices were far from mirroring it. Whereas the concept of Greater Eurasia remained on paper, the practical dimensions of connectivity linking Russia with Europe continued to flourish. The railway corridor between China and Europe has developed at a surprising pace, with the number of cargoes increasing on an annual basis. The construction of the Power of Siberia gas pipeline to China notwithstanding, the bulk of Russia’s gas export infrastructure connected Russia with its European customers. The Nord Stream 2 pipeline — with capacity bigger than its counterpart to China — seemed to reaffirm Russia’s links with Europe. In this sense, connectivity imagined in the form of Greater Eurasia stood in sharp contrast to the connectivity practiced in Russia. The logic of containment in the conceptual domain was challenged by the logic of cooperation in the practical dimension.

Russia’s war against Ukraine, fully launched in 2022, has the potential to remove this discrepancy at the cost of the existing cooperation between Russia and Europe. Faced with the Western support for Kyiv, Moscow decided to limit its connections with Europe, first and foremost by reducing and cutting off gas supplies. The gas pipeline infrastructure built over the decades has turned out to be dependent on a whim of the Kremlin. The European Union attempted to reduce oil purchases from Moscow, the Kremlin responded by reducing gas supply. At the time of writing (i.e. one year into the war), the China-Europe railway connection has not been affected by either side’s actions. However, the war led to the acceleration of efforts to bypass Russia, affecting both Russia’s role as a key transit link and Russia’s ties with Central Asian states, Kazakhstan in particular. The so-called Middle Corridor, via Central Asia, the Caspian Sea, South Caucasus and Turkey is the most probable alternative [46]. So far, the route via Russia was the cheapest and shortest one. The costs of insurance, the willingness to avoid sanctions or secondary sanctions, and the risk of Europe banning transit from Russia mean that the development of routes bypassing Russia becomes a long-term option. A prolonged conflict and the increasing scope of sanctions against Russia may lead to a complete shutdown of the railway transport from Russia, especially given the role of the Russian Railways, RZhD, in controlling the railway route.

The core of Russia’s connectivity undertakings — oil and gas pipelines — faces additional pressures. Moscow’s calls for creating additional energy transport infrastructure towards China, in the form of a trans-Mongolian gas pipeline, have not been responded to by Beijing. Other potential markets of South Korea and Japan seem closed to a possibility of new infrastructure. Thus, the plans of increasing energy exports to Asia-Pacific, envisioned by the Energy Strategy by 2035, will have to be revised accordingly.

The war in Ukraine has also impacted Russia’s position in Greater Eurasia itself, in the North-East Asia in particular. Japan and South Korea joined the Western sanctions, which may have a negative effect on the use of the Trans-Siberian corridor, especially if Russia escalates its actions in Ukraine and the Western community responds with further sanctions. So far, the war has had limited effect on Russia’s position in South-East Asia (according to Moscow’s vision, ASEAN is seen as part of Greater Eurasia). However, diminishing resources will narrow Russia’s room for manoeuvre, making the implementation of the GEP an even more distant prospect.

## Conclusions

The aim of this article has been to bring Russia into the debate on connectivity. In order to contribute to the concept’s development, it has followed the theoretical framework put forward by Gaens, Sinkkonen and Vogt [18] in the theoretical framework of this special issue and identified different logics and spheres of Russia’s connectivity strategy and practice, suggesting elaborations to the framework. The paper suggests that Russia’s take on connectivity is characterised by fluidity, with different logics and spheres dominating in different geographical spaces. In Asia, we have identified three such spaces: Russia’s Far East, the post-Soviet Central Asia, and Greater Eurasia. At the same time, the presence of contestation, containment, and coercion as logics of connectivity is easily recognisable, especially with regard to Russia’s approach to the West and its post-Soviet neighbours. In the case of Russia’s approach to China, we have observed the tensions between the attempt to exclude China similarly to Western states (containment) and to take measures to reconcile the Russian connectivity project with its Chinese counterpart (cooperation and cushioning). Finally, our analysis of the RFE serves to point out that it is fruitful to extend the analysis of connectivity to the subnational level, especially in a territorially large federal state like Russia.

The article points to the gap between Russia’s strategy and its implementation on the ground, thus affirming the findings of existing literature on the role that physical infrastructure, trade, and logistics services have played in Russian history and contemporary politics [11, 38, 44]. For example, the development of GEP according to the containment logic vis-à-vis Europe was not matched by practical connectivity links, which followed the logic of cooperation (in such areas as railway connections China-Europe and energy resources trade Russia-Europe). Additionally, Russia has engaged in the logic of copying in its connectivity projects — it was explicit in the case of the EAEU, with the treaties copied after the EU legislation and it was implicit in the case of GEP, which resembles the BRI with its open-ended aims, lack of clear boundaries, and general vagueness of the project.

Russia’s ongoing war in Ukraine has meant a major setback to Moscow’s connectivity strategy. Russia’s relations with the EU, USA, and other Western actors are now characterised by increasing *dis*connectivity, pursued primarily by those supporting Ukraine. In Central Asia, too, Russian involvement in connectivity enhancing activities is declining due to the lack of resources and lack of political will in the region, at least for now [43]. The GEP can be simply forgotten. At the same time, the war has demonstrated that connectivity cannot be undone overnight. Both China and Central Asian states seem interested in extending their connectivity projects with Russia, especially if it does not rule out the improvement of their connectivity with other actors. This leads us to suggest that Russia will be a connectivity actor of its own right for decades to come regardless of the dramatic decline of its resources and its role in the international community.

## Footnotes

1 The authors have no competing interests to declare that are relevant to the content of this article. They received no funding for conducting this study.

2 The decision to apply the theoretical framework by Gaens, Sinkkonen and Vogt [18] rather that construct a separate model for studying specifically Russia’s connectivity strategy has been informed by our desire to contribute to the development of a universal framework of connectivity. While this article maintains that the framework is a suitable tool for analysing Russia’s choices in the sphere of connectivity, its explanatory power has its limitations [Cf. 41].

3 The launch of Russia’s full-scale war in Ukraine in February 2022 is likely to yield another shift in Russia’s connectivity strategy, but its examination lies beyond the scope of this article.

4 Given the opaque nature of politics in Russia, it is methodologically challenging to offer definite explanations of the authorities’ reactions. According to an alternative interpretation, the decision not to suppress the Khabarovsk protests was informed by an assumption that the protests would evaporate on their own [10], while their suppression could spark a chain-reaction of protests across the country dangerous for the regime.

5 In addition to the five post-Soviet states, the presence of a visa-free regime has led to many Russians migrating to Mongolia, especially from the Republic of Buryatia across the border. The influx has been propelled by Mongolia’s decision to grant temporary residency permits to all applicants [29].
